# Immunotherapy and Antivascular Targeted Therapy in Patients' Treatment with Concurrent Malignant Tumors after Organ Transplantation: Opportunity or Challenge

**DOI:** 10.1155/2022/6440419

**Published:** 2022-06-02

**Authors:** Bairu Shen, Zi Guo, Peng Huang, Minghua Tan, Xiaoshen Zhang, Siyao Lin, Changshan Song, Jiaqing Wang, Minqian Huang

**Affiliations:** ^1^Thoracic Surgery, Foshan Clinical Medical School of Guangzhou University of Chinese Medicine, Guangdong Province, China; ^2^Cardiac Surgery, The First Affiliated Hospital of Jinan University, Guangdong Province, China

## Abstract

**Objective:**

To analyze the therapeutic effects and organ rejection of anti-PD-1 immunotherapy or antivascular targeting therapy on patients with combined malignancies after organ transplantation.

**Methods:**

We collected retrospective studies on “post-transplantation, cancer, immunotherapy, and vascular targeting therapy” in Embase, Wanfang database, Cochrane Library, VIP databases, CNKI, and PubMed, and the case data were organized and analyzed.

**Results:**

Data from only 40 papers met our requirements, which included 2 literature reviews, 4 original researches, and 34 case reports from 2016 to 2020. A total of 40 studies involving 66 patients were included, who were divided into 3 groups (patients using CTLA-4 inhibitors, group 1; patients who received sequential or concurrent anti-PD-1 and anti-CTLA-4 therapy, group 2; and patients using PD-1/PD-L1 inhibitors, group 3). There was no statistical difference in patients' DCR between the three groups (*P* > 0.05). Also, compared with group 2, there was no statistically significant difference in recipient organ rejection in group 1 and group 3 (*P* > 0.05). The DCR rate for antivascular targeted therapy is approximately 60%.

**Conclusions:**

Immunotherapy should be carefully selected for patients with combined malignancies after organ transplantation. Antivascular targeted therapy is one of the options worth considering; the risk of side effects of drug therapy is something that needs to be closely monitored when combined with immunotherapy.

## 1. Introduction

As transplant operation is becoming more and more advanced, the number of solid organ transplant (SOT) patients is rising these years, such as kidney recipients and liver recipients [1, 2]. Although organ transplant operation extends patients' lives, it has drawbacks like a doubled-edged sword. It has previously been observed that the immunosuppression after SOT made such kind of people to become a high incidence of cancer [3]. Previous researches have indicated that recipients had a higher risk than the general population to have diverse infection-related or unrelated malignant tumors [4, 5], especially skin cancers [6]. Nevertheless, the treatment for malignant tumors in these special patients is tricky for physicians [7], especially in chemotherapy such as immunotherapy and antivascular targeted therapy. It is said that immunotherapy is a great evolution for malignant tumor treatments, which has great curative effect on cancer patients [8, 9]. Data has shown that immunotherapy performs excellently in treating melanoma and non-small-cell lung cancer (NSCLC) [10, 11]. However, a debated question of whether immunotherapy can benefit SOT patients with malignancies is unclear nowadays, as the mechanism of action of immunotherapeutic drugs may lead to rejection of the graft in SOT patients [12]. Besides, the present randomized controlled trial (RCT) and clinical trials have not included such kind of patients for these patients are not common enough [13], which lead to a lack of data and treatment experiences for this population. Therefore, it is essential to explore if immunotherapy can benefit SOT patients with malignancies and suggest a preferable treatment for these patients. Nevertheless, there have been no studies that conduct secondary analysis of data related to immunotherapy for these patients yet, just some case reports and reviews. Most of the information is based on retrospective case studies; the experience of most physicians is not uniform as to whether immunotherapy can benefit SOT patients with malignancies and increase the risk of rejection in the recipient organ.

Antivascular targeted therapies are currently common therapeutic agents for malignancies such as lung and bowel cancer, especially in combination with chemotherapy or immunotherapy showing good disease control rates in patients lacking a mutated target. However, data on the use of such agents in SOT patients remain scarce. The potential risk of damage to the transplanted organ from antivascular targeted agents is also unclear.

Whether immunotherapy and antivascular targeted therapy are promising treatment modalities for physicians awaits detailed analysis of clinical data. Therefore, we summarized and analyzed data from studies in this population to discuss the effectiveness of immunotherapy in this patient population and the risk of organ rejection occurrence.

## 2. Methods

**Figure 1:**
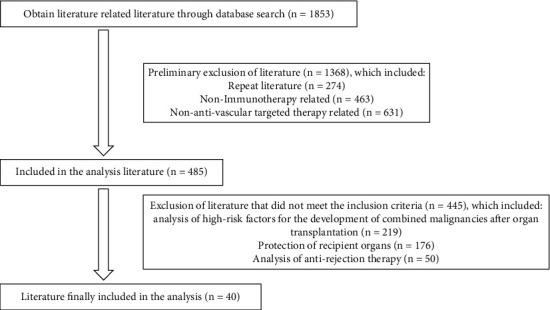
The results of the literature search for immunotherapy.

We used “post-transplantation, cancer, and immunotherapy” as search terms to search the literature information in the Wanfang database, Cochrane Library, Embase, CNKI, PubMed, and VIP database. The search indicated that there were 485 papers discussing the treatment of patients with combined malignant tumors after organ transplantation. Most of the papers focused on the high-risk factors for comorbid malignancies' development after organ transplantation, the protection of recipient organs, and the adaptation of antirejection treatment regimens. Information is in Figure 1.

**Figure 2:**
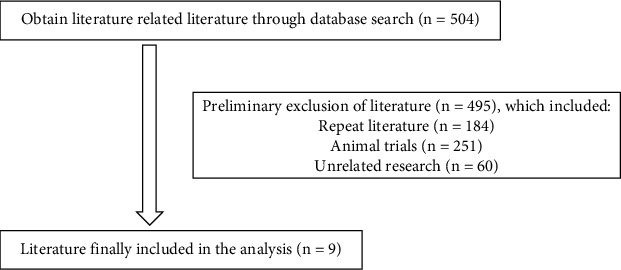
The results of a literature search for antivascular targeted therapies.

In addition, we also used “carcinoma, transplantation, apatinib, anlotinib, and bevacizumab” as search terms to search the articles about tumor antivascular targeted therapy in CNKI, PubMed, and Embase. There are 504 papers in total. Excluding articles about animal trials, repeated literatures, and unrelated research, we included 9 case reports at last for our case review about SOT patients with combined malignancies using antivascular targeted therapy. Information is in Figure 2.

The cases enrolled in the study were divided into three groups according to the treatment regimen: group 1: patients using CTLA-4 inhibitors, group 2: patients who received sequential or concurrent anti-PD-1 and anti-CTLA-4 therapy, and group 3: patients using PD-1/PD-L1 inhibitors.

## 3. Statistical Analysis

A standardized data collection template was used by us in order to extract the following information: age, transplant organs, antirejection methods, time from transplant to cancer, tumor types, immunotherapy way, outcomes of the therapy, and rejection reaction after therapy. The statistical data were expressed as percentages. The difference in patients and cancer characteristics was tested using Fischer or Pearson chi square for categories. Survival analysis was performed using the Kaplan-Meier model.

## 4. Result

### 4.1. Analysis of the General Situation of the Case Data

Data from only 40 papers met our requirements, which included 2 literature reviews, 4 original researches, and 34 case reports from 2016 to 2020. Altogether, our research identified 66 cases, from which 55 cases were included in the efficacy analysis. SOT patients receiving immunotherapy for cancer were our population of interest. Their ages fluctuated from 14 years to 85 years. 13 patients were on anti-CTLA-4 therapy, 9 patients were on sequential or concurrent anti-PD-1 and anti-CTLA-4 therapy, and 44 patients were on anti-PD-1/PD-L1 therapy. The main combined malignancy types in SOT patients were melanoma (40 cases) and hepatocellular carcinoma (HCC) (10 cases). Besides, the most common transplant was the kidney; next was the liver and heart. There were totally 11 kinds of antirejection treatment schemes (before immunotherapy). In the cases without an antirejection treatment scheme before immunotherapy, we tacitly approved the after ones as the initial ones. For the age, time from transplant to cancer groups, transplants, tumor type, and antirejection information of the three groups of patients, the differences between the groups were insignificant (*P* > 0.05), and the information was comparable. Information is in Table 1.

### 4.2. Analysis of Data on Concomitant Malignancies after Organ Transplantation Treated with Antivascular Targeted Therapy

The disease control rate (DCR) after antivascular targeted therapy was 60% (6/10), with a partial response (PR) of 30% (3/10), a complete response (CR) of 20% (2/10), and stable disease (SD) of 10% (1/10). The time from transplantation to cancer ranged from 3 months to 19 years. The tumor types were mainly metastatic carcinoma of the lung, synovial sarcoma, and colorectal cancer. Details are in Table 2.

### 4.3. Analysis of the Therapeutic Effect of Anti-PD-1/PD-L1 and Anti-CTLA-4 Immunotherapy and the Occurrence of Rejection

We divided the outcomes into two categories according to the solid tumor evaluation criteria RECIST1.1, DCR and PD. The first response after using immunotherapy was determined in our articles. There were 55 cases in total that were included in the efficacy analysis/Fisher's exact test. The result showed that the cure rate of group 1 was about 46.2%, while the cure rate of group 2 was about 37.5%, and that of group 3 was about 50%. There was no significant difference in SOT patients with malignant tumors treated with different immunotherapies (*P* ≥ 0.05). Information is shown in Table 3.

We included all the 66 patients, group 1 was 13 patients, while group 2 was 9 patients and group 3 was 44 patients. Regarding the occurrence rate of organ rejection after immunotherapy, group 1 was 23.1%, while group 2 was 44.4% and group 3 was 45.5%. We still did not see significant differences in this analysis (*P* ≥ 0.05). Information is shown in Table 4.

For the data analysis of gender, we have done subgroup analyses to explore if gender could influence curative effect or organ rejection. The differences in the gender subgroup data of the three groups were not statistically different (*P* ≥ 0.05). In addition, there was not much research data to confirm that gender was an important factor in the efficacy and target organ effects of immunotherapy. Therefore, we will not discuss gender as a factor in this article. Information is shown in Tables 5 and 6.

### 4.4. Analysis of Survival after Immunotherapy in Patients with Combined Malignancy after Organ Transplantation

**Figure 3:**
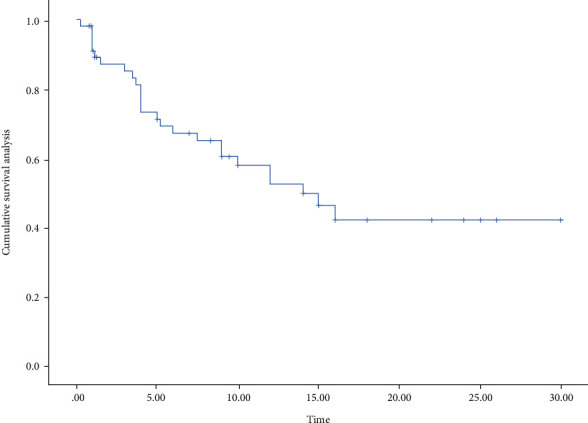
Survival analysis of immunotherapy in SOT patients.

**Figure 4:**
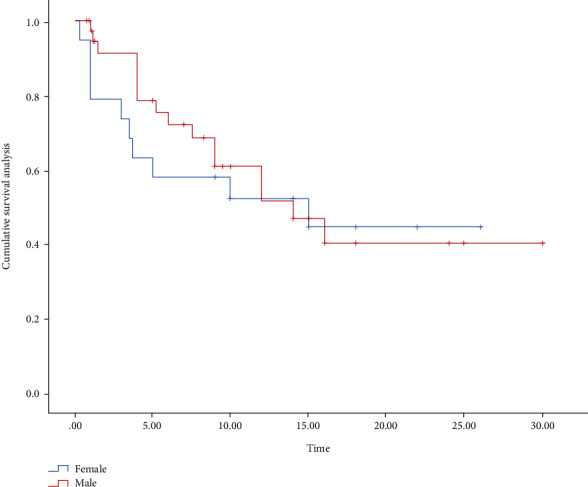
Survival analysis of gender subgroups of SOT patients.

**Figure 5:**
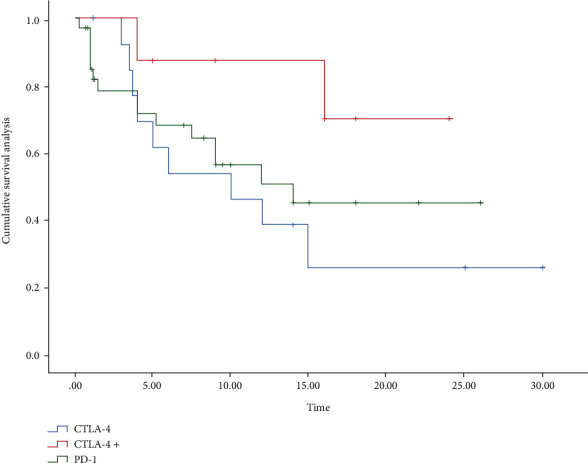
Treatment group survival analysis of SOT patients. CTLA-4: patients using anti-CTLA-4; CTLA-4+: patients who received sequential or concurrent anti-PD-1 and anti-CTLA-4 therapy; PD-1: patients using anti-PD-1/PD-L1.

Based on the data collected from previous studies, with the assistance of Stats, we performed the survival analysis in all patients (Figure 3), as well as the survival analysis in different genders and each group (Figures 4 and 5). The median survival for all patients was approximately 15 months, with approximately 16 months for men and 10 months for women and approximately 6 months for anti-CTLA-4 treatment and 14 months for anti-PD-1/PD-L1 treatment. The differences were not statistically significant between treatment modalities and between genders (*χ*^2^ = 0.191, *P* = 0.662; *χ*^2^ = 3.128, *P* = 0.209). Their overall survival (OS) was defined as a period between ICI's administration and death's time or end of the follow-up. If the death time was not available, the publication time was a substitute. What could be clearly seen in these figures was the difference in OS according to the gender group. The median overall survival (mOS) was approximately 15 months in female and 16 months in male (95% CI: 13.13-20.22).

### 4.5. Case Report of Anti-PD-1 Combined with Antivascular Targeted Therapy

**Figure 6:**
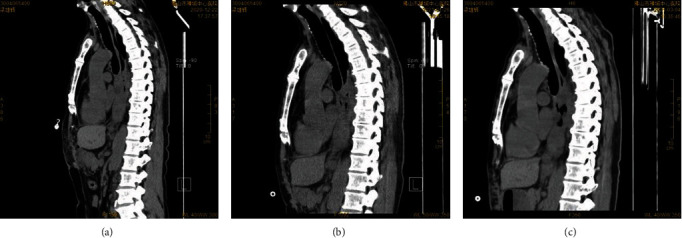
The change of esophageal tumor before and after treatment was seen as a slowly increasing tumor: (a) 23-Dec-20; (b) 26-Jan-21; (c) 5-Mar-21.

**Figure 7:**
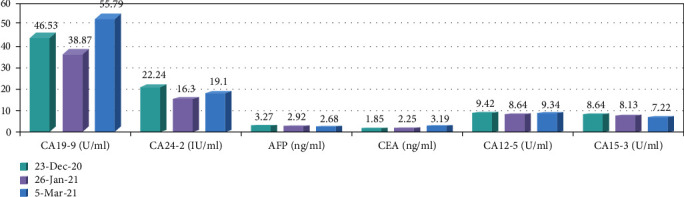
The fluctuation of tumor indexes before and after esophageal tumor treatment. CA: glycoantigens; AFP: alpha-fetoprotein; CEA: carcinoembryonic antigen.

**Figure 8:**
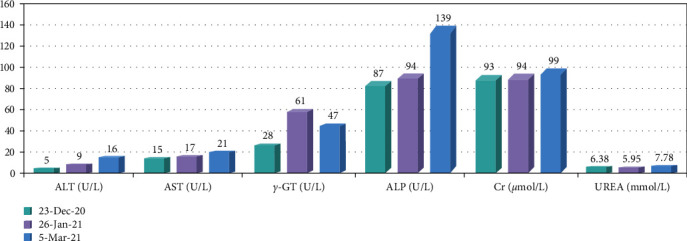
The fluctuation of liver and kidney function indicators before and after esophageal tumor treatment. ALT: alanine aminotransferase; AST: aspartate transaminase; *γ*-GT: glutamyl transpeptidase; ALP: alkaline phosphatase; Cr: creatinine.

We report a case about a SOT patient treated with anti-PD-1 for an esophageal tumor. The patient was a 51-year-old male with a previous allogeneic liver transplant for alcoholic liver disease, after which he was treated with tacrolimus and mycophenolate mofetil as a normative antirejection therapy. About 1 year later, he was diagnosed with esophageal squamous cell carcinoma through gastroscopy in December 2020. The patient's pretreatment CA19-9 and CA24-2 were significantly elevated. The genetic test report of the patient's tumor tissue suggested HER2 mutation, but the patient firmly refused treatment with trastuzumab. Since there was no evidence-based medical evidence for immunotherapy's efficacy in patients' treatment with concomitant malignancies after organ transplantation, we gave a detailed explanation of the risks of treatment to the patient and his family, after which they expressed their wish to receive immunotherapy. The patient then received camrelizumab in combination with anlotinib for 2 cycles. The liver function test was checked during the treatment, and there was no evidence of liver injury. However, a CT scan of the chest suggested a slow enlargement of the esophageal tumor. About 4 weeks after the second cycle of treatment, the patient started vomiting with dark black viscous-like liquid; he suddenly died of cardiac arrest at home 2 hours later. Information is shown in Figures 6–8.

## 5. Discussion

The choice of treatment modality for combined malignancy after organ transplantation is a difficult problem for oncologists and surgeons. Because of the need to protect the recipient organ, the choice of treatment modality to kill tumor cells is difficult. There are no treatment recommendations in the drug formulary for this particular patient. Clinical trials usually exclude the enrollment of such special patients. Analysis of case information from retrospective studies is an important way to understand how we treat patients with this particular type of tumor. Moreover, data from some of the real-world studies suggest that anti-PD-1 seems to have a more pronounced rejection response, and therefore, anti-CTLA-4 immunotherapy is recommended as a treatment for patients from the perspective of transplant organ protection [23, 24]. This differs from the results of our pooled analysis. We pooled the clinical data of 66 patients, and the results showed that anti-PD-1/PD-L1 immunotherapy was slightly more effective than anti-CTLA-4, but the difference between the two groups of patients was insignificant, so it cannot yet be considered that anti-PD-1/PD-L1 immunotherapy is superior to anti-CTLA-4 immunotherapy. The use of anti-PD-1/PD-L1 and anti-CTLA-4 before and after did not significantly increase the therapeutic effect compared with the use of the drugs alone. Although the two survival curves are clearly separated, the current data do not yet clearly see a significant survival benefit, with the small sample size in the combination therapy group being the main reason. And the different drug combinations did not result in a significant survival benefit. However, the potential risk of toxic side effects on patients increases significantly with the increased variety of immunotherapeutic agents used [25, 26], and therefore, the use of anti-PD-1/PD-L1 immunotherapy and anti-CTLA-4 immunotherapy before and after this treatment modality is not a highly recommended approach.

Although anti-PD-1/PD-L1 immunotherapy rejection is higher than anti-CTLA-4 immunotherapy, the difference is not statistically significant, so it is not very certain that anti-PD-1/PD-L1 rejection is stronger than anti-CTLA-4. The PD-1 pathway has more research data in animal models of immune exclusion [24]; however, the relationship between the CTLA-4 pathway and immune rejection needs more data to be confirmed, as the CTLA-4 pathway crosses over with the PD-1 pathway at many points [27, 28]. More than one author has suggested that there are differences between anti-CTLA-4 and anti-PD-1. Studies have shown that PD-1 inhibitors are more effective than anti-CTLA-4 in such an exceptional group of patients [29, 30]. However, in our study, such a conclusion could not be confirmed for SOT patients. We will discuss the reasons for no significant difference between different immunotherapies in terms of efficacy and incidence of rejection by the characteristics.

There are some commonalities and differences between the PD-1 pathway and CTLA-4 pathway. Effector T cells' activity can be increased (by PD-L1 combined with anti-PD-1), which inhibits PD-1 combined with PD-L1 on T cells (thymus-dependent lymphocyte cells), leading to enhanced antitumor immune-mediated responses [9, 31–33]. Anti-CTLA-4 can also activate T cells by combination with CTLA-4, which may compete with CD28 and bind to CD80/86 to inhibit T cell activation [34–39]. CTLA-4 is said to be responsible for the activation of T cells during their initial phase, whereas PD-1 is thought to regulate effector T cells' function in tissues and tumors [40]. On the other hand, they have 3 common intersections in signaling pathways. First, like CTLA-4, PD-1 is highly expressed in Treg cells (regulatory T cells) [41], and they both increase Treg cells' immunosuppressive function [42, 43], where Foxp-3 is highly expressed. Second, anti-CTLA-4 and anti-PD-1 increase IL-2 to activate donor-specific T cells, cytotoxicity, and protection from apoptosis by allogeneic reactivity [27]. Finally, anti-CTLA-4 and anti-PD-1 induce phosphorylation of the T cell receptor (TCR) signaling pathway [30], reducing activation signals downstream of the TCR pathway and preventing T cell activation. Studies have also found synergy between anti-PD-1 and anti-CTLA-4 [44, 45]. The combination of anti-PD-1 or anti-CTLA-4 may show better efficacy than alone [46, 47]. Patients using nivolumab in combination with ipilimumab may respond more quickly [48, 49]. However, in our study, although group 2, which included only 1 patient using a PD-1 inhibitor in combination with CTLA-4 inhibitor, showed good results, this is not strong evidence to support this conclusion.

However, due to the lack of cases and controlled trials, it is still difficult to confirm that nivolumab has a higher response rate than ipilimumab or that the combination of these two drugs is better than alone. On the other hand, for immunotherapy in normal tumor patients, D'Angelo et al. [50] showed no significant difference between the use of nivolumab alone and the use of nivolumab in combination with ipilimumab [51], whose study showed the same results as our study. Therefore, we hypothesize that the efficacy of immunotherapy is not as drug selective as in the normal population.

Antivascular targeted therapy is currently one of the important modalities in the treatment of malignant tumors and has shown good therapeutic effects on lung and intestinal cancers. Available data suggest that there is a synergistic effect of antivascular targeted therapy and immunotherapy [52–55], and some studies suggest that antivascular targeted therapy can reverse PD-1 resistance to some extent [56]. Retrospective data analysis suggests that antivascular targeted therapy has also shown good disease control rates in patients with specific tumors after organ transplantation. Data on antivascular targets combined with anti-PD-1 immunotherapy in organ transplant are lacking [57]. Our study data show that antivascular targeted therapy has a DCR rate of approximately 60% and does not significantly increase the risk of organ rejection, and although the data are dominated by a limited number of empirical cases, it is still one of the options worthy of consideration by physicians. In the case we reported, there was no serious organ rejection with this treatment modality; however, the esophageal tumor was not well controlled. Hemorrhage is a serious adverse effect of antivascular targeted therapy, but available data show that combined immunotherapy does not increase the risk of bleeding or grade 3-4 adverse effects are tolerable [51]. The cause of the patient's upper gastrointestinal bleeding was presumably related to tumor rupture and was not drug-induced bleeding.

Our study's limitation is that most of the study data are from retrospective studies because the number of cases of patients with this particular malignancy is lacking. We were only able to analyze the relationship between treatment and prognosis from a limited amount of data, but we will continue to focus on this area of research.

For patients after organ transplantation, our main concern is the risk of possible rejection after oncology treatment, whether it is immunotherapy or antivascular targeted therapy. Although antivascular targeted therapy may theoretically have a synergistic effect with immunotherapy as a mechanism for releasing immunosuppression, the potential risk of rejection of transplanted organs remains. However, as shown in our summary of cases, the number of patients with SOT combined with malignancy who develop rejection with antivascular targeted therapy is modest. Among the rejection phenomena occurring in allografts, there are also pathological changes in the microvasculature [58]. Researchers have found increased VEGF expression in acute rejection and chronic rejection [59]. On the one hand, VEGF is a key mediator of remodeling of blood vessels as a consequence of injury in rejection [60]. On the other hand, VEGF may act as a proinflammatory cytokine to activate effective T cells [61, 62], thus promoting and maintaining the rejection response [63, 64]. CD8+ T cells kill their target cells by releasing cytotoxic molecules onto the graft [65], and VEGF-expressing T cells may be increased in allografts [61, 62]. Overexpression of VEGF may lead to chronic rejection and vasculopathy of the graft [66]. Furthermore, it was found that blockade of VEGF/VEGFR controlled the progression of acute rejection and pathological changes in grafted vessels in humanized mouse models [61, 62, 67]. Studies have also shown that VEGF inhibitors can prevent rejection of transplantation [68].

## 6. Conclusions

The effect of anti-PD-1 or anti-CTLA-4 immunotherapy did not reflect a significant difference in patients with combined malignancies after organ transplantation, and even sequential dosing did not show a better treatment effect. Study data showed a trend toward better transplant organ protection with anti-CTLA-4 therapy.

However, a significant difference was not shown by statistical analysis. Therefore, regardless of the immunotherapy modality, it should be chosen carefully for this particular group of patients, as our treatment experience is very limited. Antivascular targeted therapy is a treatment option worth considering because of the good DCR rates, although study data are limited. However, combination immunotherapy still requires careful consideration of treatment complications and the risk of rejection of transplanted organs. This is only a small retrospective analysis of the data, and more prospective studies are needed to confirm the data.

## Figures and Tables

**Table 1:** Analysis of general information of the patients.

	Total	Group 1	Group 2	Group 3	*P*_1_	*P*_2_
Patients (*n*)	66	13	9	44		
Age group (y)	66	13	9	44		
≥50	56	11	8	37	1.000	1.000
<50	10	2	1	7		
Time from transplant to cancer groups (y)	61	13	8	40		
≥5	33	7	5	21	0.897	1.000
<5	28	6	3	19		
Transplants	66	13	9	44		
Kidney	42	9	6	27	0.190	0.776
Liver	18	3	1	14		
Heart	4	1	1	2		
Other^1^	2	0	1	1		
Tumor type	66	13	9	44		
Melanoma	40	13	8	19	0.057	0.409
SCC	6	0	1	5		
HCC	10	0	0	10		
Other^2^	10	0	0	10		
Antirejection	65	13	9	43		
Glu+CNIs+AP	10	2	2	6	0.886	0.722
Glu+CNIs	17	2	4	11		
CNIs	12	1	1	10		
CNIs+AP	8	4	1	3		
mTOR inhibitor	3	1	0	2		
mTOR inhibitor+AP	3	1	0	2		
mTOR inhibitor+Glu+AP	2	0	1	1		
Glu+AP	3	1	0	2		
Glu	2	1	0	1		
mTOR inhibitor+Glu	3	0	0	3		
Other^3^	2	0	0	2		

Glu: glucocorticoids; CNIs: calcineurin inhibitors; AP: antiproliferative drugs; SCC: skin squamous cell carcinoma; HCC: hepatocellular carcinoma; other^1^: cornea, heart, and kidney; other^2^: NSCLC, Merkel cell carcinoma, epidermoid carcinoma, and metastatic adenocarcinoma of the duodenum; other^3^: antilymphocyte antibody+glucocorticoids+calcineurin inhibitors+antiproliferative drugs; y: year. *P*_1_: group 2 compared with group 3; *P*_2_: group 2 compared with group 1.

**Table 2:** Antivascular targeting therapy in patients with combined malignancy after organ transplantation.

Date	Author	Age	Gender	Protopathy	Allograft	Time from transplant to cancer	Tumor type	ISD	OR	Antitumor scheme	Tumor remission
2015	Fatma Yalcin Müsri et al. [14]	45, 54	Male	Renal failure	Kidney	120,228 months	Colorectal cancer	EVE+TAC	NO	FOLFIRI + BEV∗5 cycles	PD
2017	Wu et al. [15]	61	Female	End-stage renal disease	Kidney	60 months	Relapse ureter UC	Prednisone+TAC+MMF	NO	Pembrolizumab + BEV∗11 cycles	PD
2018	Yu et al. [16]	36	Male	HCC	Liver	6.5 months	Lung metastases	TAC+Sir	NO	Apatinib∗10 wee	PR
2020	Zhuang et al. [17]	54	Male	HCC	Liver	3 months	Lung metastases	Low-dose TAC (trough level, 4-6 ng/mL)	NO	Regorafenib, anlotinib, apatinib, lenvatinib	SD
2020	Zhang et al. [18]	56	Male	HCC	Liver	3 months	Leiomyosarcoma	Basiliximab∗1 dose methylprednisolone∗6 days (TAC + MMF)∗1 year	NO	Apatinib	PD
2020	Onodera et al. [19]	67	Male	Chronic renal failure	Kidney	132 months	Colorectal cancer	TAC+MMF	NO	mFOLFOX6∗2 cycles, then BEV + mFOLFOX6∗16 cycles, respectively, after 6 cycles, discontinued BEV	NA
2021	Kasherman et al. [20]	44	Female	IgA nephropathy	Kidney	36 months	HGSOC	TAC	NO	Paclitaxel with BEV	PR
2021	Khaled et al. [21]	35	Male	Fibrolamellar HCC	Liver	48 months	HCC		NO	Atezolizumab + BEV∗6 months	PR
2021	Zhang et al. [22]	43	Male	NA	Kidney	9 months	Synovial sarcoma	NA	NO	Anlotinib	CR
2021	Zhang et al. [22]	33	Male	NA	Kidney	13 months	Synovial sarcoma	NA	NO	Anlotinib	CR

Aza: azathioprine; HGSOC: high-grade serous ovarian carcinoma; CRC: colorectal cancer; LT: liver transplant; CSA: cyclosporin A; PFS: progression-free; EVE: everolimus; UC: urothelium carcinoma; ISD: immunosuppressive drugs; MMF: mycophenolate mofetil; TAC: tacrolimus; Sir: sirolimus; BEV: bevacizumab; CR: complete response; PR: partial response; SD: stable disease; PD: progressive disease; NA: not available. The DCR is the percentage of patients whose tumors shrank or stabilized and remained for a certain period of time and includes cases in CR, PR, and SD.

**Table 3:** Chi-squared test of the efficacy of immunotherapy.

	DCR	PD	Total	*P*	*χ* ^2^
Group 2	3	5	8	0.808	0.059
Group 3	17	17	34		
Total	20	22	42		
Group 1	6	7	13	0.813	0.056
Group 3	17	17	34		
Total	23	24	47		
Group 1	6	7	13	1.000	—
Group 2	3	5	8		
Total	9	12	21		

**Table 4:** Chi-squared test of organ rejection.

	Yes	No	Total	*P*	*χ* ^2^
Group 2	4	5	9	1.000	0.000
Group 3	20	24	44		
Total	24	29	53		
Group 1	3	10	13	0.148	2.088
Group 3	20	24	44		
Total	23	34	57		
Group 1	3	10	13	0.376	—
Group 2	4	5	9		
Total	7	15	22		

**Table 5:** Subgroup analyses for gender and curative effect.

		DCR	PD	Total	*P*	*χ* ^2^
Male	Group 1	4	2	6	0.286	—
	Group 2	2	5	7		
	Total	6	7	13		
	Group 2	2	5	7	0.672	—
	Group 3	12	15	27		
	Total	14	20	34		

Female	Group 1	2	5	7	0.375	—
	Group 2	1	0	1		
	Total	3	5	8		
	Group 2	1	0	1	1.000	—
	Group 3	5	2	7		
	Total	6	2	8		

Total	Group 1	6	7	13	1.000	—
	Group 2	3	5	8		
	Total	9	12	21		
	Group 2	3	5	8	0.808	0.059
	Group 3	17	17	34		
	Total	20	22	42		

**Table 6:** Subgroup analyses for gender and organ rejection.

		Yes	No	Total	*P*	*χ* ^2^
Male	Group 1	1	5	6	0.301	—
	Group 2	4	4	8		
	Total	5	9	14		
	Group 2	4	4	8	1.000	0.000
	Group 3	15	19	34		
	Total	19	23	42		

Female	Group 1	2	5	7	1.000	—
	Group 2	0	1	1		
	Total	2	6	8		
	Group 2	0	1	1	1.000	—
	Group 3	5	5	10		
	Total	5	6	11		

Total	Group 1	3	10	13	0.376	—
	Group 2	4	5	9		
	Total	7	15	22		
	Group 2	4	5	9	1.000	0.000
	Group 3	20	24	44		
	Total	24	29	53		

## Data Availability

The data for these findings are available in the manuscript.
